# Evaluation of Procalcitonin Use in Patients Discharged from the Emergency Department with Acute Respiratory Infection

**DOI:** 10.1017/ash.2021.62

**Published:** 2021-07-29

**Authors:** Garrett Fontaine, David Banach, Jeffrey Aeschlimann

## Abstract

**Background:** Acute respiratory infections (ARIs) contribute significantly to inappropriate antimicrobial prescription. The rate of such prescriptions in US emergency departments (EDs) has remained stable over time. The use of procalcitonin (PCT) testing has been shown to lower risk of mortality and to reduce antibiotic consumption. It also has the potential to aid ED physicians in stratifying ARI patients who may require antibiotics but do not require hospital admission. In this study, we described the characteristics and proportion of antibiotic prescription in patients evaluated in and discharged from the ED with ARI. **Methods:** We performed a retrospective chart review of patients diagnosed with ARI and discharged from a single academic ED between January 2018 and January 2020. We compared those for whom a PCT test was ordered to those without a PCT test ordered at ARI diagnosis. Charts were reviewed until there were 110 subjects in each of the 2 study groups. The main outcome variable was receipt of an antibiotic prescription. The χ^2^ test was used to compare the proportion of patients who received an antibiotic prescription, demographics, and clinical characteristics between the 2 groups. The Mann-Whitney *U* test was used to compare the distribution of ages between the 2 groups. **Results:** Among patients in the PCT group, 87 (79.0%) received antibiotics versus 69 (62.7%) in the non-PCT group (*P* ± 18.8 vs 52.7 years ± 17.6; *P* = .0002); more likely to have preexisting heart and lung disease (28.2% vs 15.5%; *P* = .02); more often male (58.2% vs 40%; p < 0.01); had more subjective fevers (47.3% vs 33.6%, p=0.04), sputum production (49.1% vs 28.2%, p < 0.01), and nausea (17.3% vs 8.2%, p=0.04). PCT results were low (≤0.25) in 82.7% (91) of patients, of whom 70.3% (64) received antibiotics. **Conclusions:** Patients for whom PCT testing was ordered were older, had more underlying conditions and increased severity of illness. This finding may reflect that PCT testing was more likely to be ordered in patients at risk of severe infection but not requiring admission. The proportion of antibiotics prescriptions was higher for patients who had a PCT test. For patients with a low PCT result, the proportion of patients prescribed antibiotics was high. This finding may suggest that clinical characteristics were more influential than PCT result in the decision to prescribe antibiotics. More research is needed on the role of PCT testing in antibiotic prescription decisions for patients presenting to the ED with ARI.

**Funding:** No

**Disclosures:** None

